# Long noncoding AGAP2-AS1 is activated by SP1 and promotes cell proliferation and invasion in gastric cancer

**DOI:** 10.1186/s13045-017-0420-4

**Published:** 2017-02-16

**Authors:** Fuzhen Qi, Xianghua Liu, Hao Wu, Xiang Yu, Chenchen Wei, Xiaodan Huang, Guozhong Ji, Fengqi Nie, Keming Wang

**Affiliations:** 1 Department of Hepatopancreatobiliary Surgery, Huai’an First People’s Hospital, Nanjing Medical University, Huai’an City, People’s Republic of China; 2 Department of Biochemistry and Molecular Biology, Nanjing Medical University, Nanjing, People’s Republic of China; 3 Department of Oncology, First Affiliated Hospital, Nanjing Medical University, Nanjing, People’s Republic of China; 4 Department of General Surgery, The Affiliated Yantai Yuhuangding Hospital of Qingdao University Medical College, Yantai, People’s Republic of China; 5 Department of Oncology, Second Affiliated Hospital, Nanjing Medical University, Nanjing, People’s Republic of China; 6 Department of Digestive Endoscopy and Medical Center for Digestive Diseases, Second Affiliated Hospital, Nanjing Medical University, Nanjing, 210011 People’s Republic of China

**Keywords:** lncRNA, AGAP2-AS1, Proliferation, Migration, Invasion, P21, E-cadherin

## Abstract

**Background:**

Long noncoding RNAs (lncRNAs) have emerged as important regulators of tumorigenesis and cancer progression. Recently, the lncRNA AGAP2-AS1 was identified as an oncogenic lncRNA in human non-small cell lung cancer (NSCLC) and its elevated expression was linked to NSCLC development and progression. However, the expression pattern and molecular mechanism of AGAP2-AS1 in gastric cancer (GC) have not been characterized.

**Methods:**

Bioinformatic analysis was performed to determine AGAP2-AS1 expression levels in the GC and normal tissues using gene profiling data from the Gene Expression Omnibus. Quantitative real-time polymerase chain reaction was used to validate AGAP2-AS1 expression in the GC tissues/cell lines compared with that in the adjacent nontumorous tissues/normal epithelial cells. Loss- and gain-of-function approaches were performed to investigate the effect of AGAP2-AS1 on GC cell phenotypes. The effect of AGAP2-AS1 on cell proliferation was evaluated by MTT, colony formation, flow cytometry, and in vivo tumor formation assays. The effects of AGAP2-AS1 on cell migration and invasion were examined using Transwell assays. Chromatin immunoprecipitation, luciferase reporter assays, RNA pull-down, and RNA immunoprecipitation were used to investigate the factors involved in AGAP2-AS1 dysregulation and the mechanism of action of AGAP2-AS1 in the GC cells.

**Results:**

AGAP2-AS1 was highly expressed in the GC tissues and cell lines, and patients with higher AGAP2-AS1 expression had a poorer prognosis and shorter overall survival. Furthermore, knockdown of AGAP2-AS1 significantly inhibited GC cell proliferation, migration, and invasion in vitro and tumor growth in vivo. AGAP2-AS1 overexpression promoted cell growth and invasion. In addition, the transcription factor SP1 activated AGAP2-AS1 expression in the GC cells. AGAP2-AS1 functions as an oncogenic lncRNA by interacting with LSD1 and EZH2 and suppressing CDKN1A (P21) and E-cadherin transcription.

**Conclusions:**

Taken together, these findings imply that AGAP2-AS1 upregulated by SP1 plays an important role in GC development and progression by suppressing P21 and E-cadherin, which suggests that AGAP2-AS1 is a potential diagnostic marker and therapeutic target for GC patients.

**Electronic supplementary material:**

The online version of this article (doi:10.1186/s13045-017-0420-4) contains supplementary material, which is available to authorized users.

## Background

Gastric cancer (GC) is still one of the most common malignancies, and one of the leading causes of cancer-related death worldwide [1, 2]. Although numerous efforts have been made to improve the diagnosis and survival of GC patients, this disease remains a major challenge due to the limited therapeutic options, tumor metastasis, and recurrence [3]. Despite advances in our understanding of the pathology of GC and the improvement of individualized treatment, the 5-year overall survival (OS) rate of GC patients is still lower than 30% due to more than half of patients already being at a progressive stage when diagnosed [4, 5]. It is thus essential to identify new regulators involved in GC, and there is an urgent need to develop novel diagnostic markers and effective therapeutic targets for GC patients.

In recent years, the improvement of RNA sequencing techniques and bioinformatics methods has led to the sequencing of the human genome and the ENCODE project [6]. As a result, the GENCODE annotation revealed that less than 3% of the human genome consists of protein-coding genes, while the majority of the rest transcribes into noncoding transcripts [7]. Increasing evidence has revealed that these noncoding RNAs such as miRNAs play critical roles in human cancer development [8]. Long noncoding RNAs (lncRNAs) are newly identified members of the noncoding RNA family, which are greater than 200 nucleotides (nt) in length and lack protein-coding ability [9]. It has been documented that lncRNAs participate in diverse biological processes, including X-chromosome imprinting, chromatin remodeling, RNA alternative splicing and decay, cell differentiation, cell fate control, cancer cell metastasis, and drug resistance [10]. Notably, numerous studies have linked lncRNA dysregulation with human diseases, especially cancer [11]. lncRNAs have thus been highlighted as critical regulators of tumorigenesis and cancer progression, and numerous lncRNAs have been found to function as oncogenes, tumor suppressors, or both depending on the circumstances [12].

Recently, several GC-associated lncRNAs have been characterized, and their biological function and underlying mechanisms have been documented, such as ZFAS1 [13], HOTAIR [14], HOXA-AS2 [15], and MEG3 [16]. In addition, the overexpression of LINC00152 was shown to promote GC cell proliferation and accelerate cell cycle progression by interacting with EZH2 and thereby suppressing the transcription of CDKN2B and P21 [17]. Another lncRNA termed BC032469 was also shown to be upregulated in the GC tissues and to function as competing endogenous RNA (ceRNA) to antagonize the miR-1207-5p suppression of hTERT, which promotes cell growth [18]. Moreover, low expression of the lncRNA LINC00261 was shown to be associated with a poor prognosis of GC patients, and overexpressed LINC00261 was found to suppress GC cell metastasis by affecting the epithelial–mesenchymal transition [19]. Although several lncRNAs with oncogenic or cancer-suppressive functions have been identified in GC, it remains unclear whether other lncRNAs are also involved in GC tumorigenesis and progression. Therefore, it is critically important to investigate other GC-associated lncRNAs and elucidate their biological consequences in order to understand the pathogenesis of GC.

AGAP2-AS1, an antisense lncRNA transcribed from a gene located at 12q14.1, which is 1567 nt in length, has been found to be overexpressed in human non-small cell lung cancer (NSCLC). A recent study showed that increased AGAP2-AS1 promotes cell proliferation in NSCLC by suppressing the transcription of its targets KLF2 and LATS2 [20]. However, the expression pattern, biological function, and underlying mechanism of AGAP2-AS1 in human GC remain unclear. In this study, we identified that AGAP2-AS1 was highly expressed in the GC tissues and cells and that higher AGAP2-AS1 expression was related to poor patient prognosis. We also investigated the contributions of AGAP2-AS1 to GC tumorigenesis and progression by applying loss- or gain-of-function assays. Moreover, we used RNA immunoprecipitation (RIP), chromatin immunoprecipitation (ChIP), and luciferase reporter assays to investigate the factors involved in AGAP2-AS1 dysregulation and characterize the mechanism by which AGAP2-AS1 regulates its targets in the GC cells. Taken together, the obtained findings may provide new insights into the critical role of the lncRNA AGAP2-AS1 in human GC tumorigenesis and progression.

## Methods

### Tissue samples and cell lines

Fifty paired GC and adjacent nontumor tissue samples were obtained from patients who had been diagnosed with GC based on a histopathological evaluation and undergone surgery at the Second Affiliated Hospital of Nanjing Medical University between 2011 and 2012. Clinicopathological characteristics, including gender, age, tumor-node-metastasis (TNM) staging, and tumor size, were recorded. These patients had not undergone any local or systemic treatment before surgery. All tissue samples were immediately snap-frozen in liquid nitrogen and stored at −80 °C until used for RNA extraction. This study was approved by the Research Ethics Committee of Nanjing Medical University, China. Written informed consent was obtained from all patients. Five GC cell lines (BGC823, SGC7901, AGS, MGC803, and MKN45) and one normal gastric epithelial cell line (GES1) were purchased from the Shanghai Cell Bank of the Chinese Academy of Sciences (Shanghai, China). The BGC823, MGC803, and MKN45 cells were cultured in RPMI 1640; the SGC7901 and AGS cells were cultured in DMEM (GIBCO-BRL) supplemented with 10% fetal bovine serum (FBS), 100 U/ml penicillin, and 100 mg/ml streptomycin (Invitrogen, Carlsbad, CA, USA) at 37 °C in 5% CO_2_.

### RNA extraction and qRT-PCR assays

The total RNA of the tissue samples and cells was isolated using TRIzol Reagent (Invitrogen, Carlsbad, CA, USA), in accordance with manufacturer’s instructions. Then, 1 μg of total RNA was reverse-transcribed in a volume of 20 μl using random and oligo dT primers under standard conditions, in accordance with the instructions of the PrimeScript RT Kit (TaKaRa, Dalian, China). For qRT-PCR assays, we used SYBR Premix Ex Taq (TaKaRa, Dalian, China) to determine the expression levels of AGAP2-AS1 and its targets, in accordance with manufacturer’s instructions. The expression data of AGAP2-AS1 were normalized to the expression of glyceraldehyde-3-phosphate dehydrogenase (GAPDH). The primer sequences are listed in Additional file 1: Table S1.

### Cell transfection

The human AGAP2-AS1 cDNA sequence was synthesized and then ligated into the pCDNA3.1 vector (Invitrogen, Shanghai, China). AGAP2-AS1 stealth siRNAs were purchased from Invitrogen, and AGAP2-AS1 short hairpin RNA oligos were synthesized, annealed, and ligated into the shRNA vector. Plasmid vectors (pCDNA-AGAP2-AS1, sh-AGAP2-AS1, and empty vector) for transfection were prepared using Midiprep kits (Qiagen, Hilden, Germany). si-AGAP2-AS1 or si-NC was transfected into the BGC823 or AGS cells growing on six-well plates using RNAiMAX (Invitrogen, Shanghai, China), in accordance with manufacturer’s instructions. The siRNA and shRNA sequences are listed in Additional file 1: Table S1. Forty-eight hours after transfection, the cells were harvested for qRT-PCR or Western blot analysis. The BGC823 cells stably transfected with sh-AGAP2-AS1 or empty vector were selected by G418, and then the cloned cells were collected for an in vivo tumorigenesis experiment.

### Cell proliferation assays

The viability of the BGC823 and AGS cells after si-AGAP2-AS1 (3000 cells/well) or negative control treatment when grown on 96-well plates was monitored using Cell Proliferation Reagent Kit I (MTT) (Roche Applied Science) and EdU assay kit (Life Technologies Corporation, Carlsbad, CA, USA), following manufacturer’s protocol. For each treatment group, wells were assessed in triplicate. For the colony formation assay, the BGC823 and AGS cells transfected with sh-AGAP2-AS1 or empty vector were cultured in six-well plates. After 2 weeks, the cells were fixed with methanol for 30 min. After washing, the cells were stained with 0.5% crystal violet for 30 min and counted. For cell cycle analysis, the BGC823 and AGS cells transfected with si-AGAP2-AS1 or negative control were harvested 48 h after transfection by trypsinization. Then, these cells were stained with propidium iodide (PI) using the CycleTEST™ PLUS DNA Reagent Kit (BD Biosciences, Franklin Lakes, NJ, USA), in accordance with manufacturer’s protocol, and analyzed by FACScan. The proportions of the cells in the G_0_/G_1_, S, and G_2_/M phases were counted. All experiments were performed in quadruplicate.

### Apoptosis assays

The BGC-823 and AGS cells transfected with si-AGAP2-AS1 or si-NC were harvested 48 h after transfection by trypsinization. After double staining with FITC-annexin V and PI, the cells were analyzed with a flow cytometer (FACScan®; BD Biosciences) equipped with CellQuest software (BD Biosciences, Franklin Lakes, NJ, USA).

### Cell migration and invasion assays

Twenty-four-well Transwell chambers with 8-μm pore size polycarbonate (Corning Incorporated, Corning, NY, USA) were used for cell migration and invasion assays. For invasion assays, the top side of the membrane was coated with Matrigel (BD Biosciences, Franklin Lakes, NJ, USA), and then 1 × 10^5^ cells (in each well) in serum-free DMEM or RPMI 1640 medium were seeded on the chambers. DMEM or RPMI 1640 containing 10% FBS was added to the wells under the chamber. For migration analysis, 5 × 10^4^ cells (in each well) in serum-free DMEM or RPMI 1640 medium were seeded on the chambers without Matrigel. After 24 h of incubation, cotton swabs were used to remove the cells inside the upper chamber, while the cells on the other side of the membrane surface were fixed and stained with 0.5% crystal violet solution. Five random fields were counted in each well.

### Tumor formation assay

Four-week-old athymic BALB/c nude mice were maintained under specific pathogen-free conditions. The mice were manipulated in accordance with the protocols approved by the Shanghai Medical Experimental Animal Care Commission. The BGC823 cells stably transfected with sh-AGAP2-AS1 or empty vector were harvested and washed with PBS. Then, 1 × 10^7^ cells were subcutaneously injected into the ventral side of each mouse for tumor formation assays. The tumor volumes were examined every 3 days and calculated using the following equation: *V* = 0.5 × D × d^2^ (V, volume; D, longitudinal diameter; and d, latitudinal diameter). This study was carried out in strict accordance with the recommendations in the Guide for the Care and Use of Laboratory Animals of the National Institutes of Health. The protocol was approved by the Committee on the Ethics of Animal Experiments of Nanjing Medical University.

### RNA immunoprecipitation (RIP)

RIP assay was used to determine whether AGAP2-AS1 interacts with or binds to RNA-binding proteins (EZH2, SUZ12, and LSD1) in the human GC cells. The EZ-Magna RIP kit (Millipore, Billerica, MA, USA) was used to conduct the RIP experiment, following manufacturer’s protocol. The BGC-823 and AGS cells were lysed using complete RIP lysis buffer; then, the extract was incubated with magnetic beads conjugated with EZH2, SUZ12, and LSD1antibodies or control IgG (Millipore) for 6–8 h at 4 °C. Next, the beads were washed with washing buffer and incubated with proteinase K at 55 °C for 30 min to remove the proteins. Finally, purified RNA was reverse-transcribed into cDNA and subjected to qRT-PCR analysis to determine the presence of AGAP2-AS1 using specific primers. EZH2 (Cat.No.17-662), SUZ12 (Cat.No.03-179), LSD1 (Cat.No.17-10531), CoREST (Cat.No.07-455), and HuR (Cat.No.03-102) antibodies were purchased from EMD Millipore.

### Luciferase reporter assays

The JASPAR (http://jaspar.genereg.net/) online database was used to predict potential transcription factor binding sites at the AGAP2-AS1 promoter regions, and several SP1 binding motifs were identified. The AGAP2-AS1 promoter region (2000 bp) was then synthesized and inserted into a pGL3-basic vector (Promega, Madison, WI, USA). The successful integration of this sequence into the vector was verified by sequencing. The Dual-Luciferase Assay Kit was used to assess luciferase activities, following manufacturer’s protocol.

### Chromatin immunoprecipitation (ChIP) assays

The EZ-Magna ChIP kit (EMD Millipore) was used to conduct the ChIP assays. In accordance with manufacturer’s protocol, the BGC-823 and AGS cells were fixed with 4% paraformaldehyde and incubated with glycine for 10 min to generate DNA–protein cross-links. Then, the cells were lysed with Cell Lysis Buffer and Nuclear Lysis Buffer and sonicated to generate chromatin fragments of 200–300 bp. Next, the lysates were immunoprecipitated with Magnetic Protein A Beads conjugated with EZH2- (Cat. 17-662, EMD Millipore), H3K27me3- (Cat. 17-622, EMD Millipore), LSD1- (Cat. 17-1053, EMD Millipore), or H3K4me2-specific antibodies (Cat. 17-677, EMD Millipore), or IgG as a control. Finally, the precipitated DNA was analyzed by qRT-PCR.

### Subcellular fractionation analysis

The distribution of AGAP2-AS1 in the nuclear and cytosolic fractions of the BGC823 and AGS cells was assessed using the PARIS Kit (Life Technologies), in accordance with manufacturer’s instructions.

### Western blot assays

RIPA extraction reagent (Beyotime, Beijing, China) supplemented with protease inhibitor cocktail (Roche, CA, USA) was used to lyse the BGC823 and AGS cells and extract the protein. Then, 40 μg of protein was separated by 8–12% SDS-polyacrylamide gel electrophoresis (SDS-PAGE) and transferred to 0.22-μm Pvdf membranes (Millipore). The membranes were sealed using 5% BSA in PBS and incubated with P21, E-cadherin, or GAPDH antibodies (all from Cell Signaling Technology). ECL chromogenic substrate was quantified by densitometry (Quantity One software; Bio-Rad).

### RNA pull-down assays

AGAP2-AS1 RNA was transcribed in vitro using T7 RNA polymerase, and pcDNA-AGAP2-AS1 vector was used as template (Ambio Life). The transcribed RNA was purified using the RNeasy Plus Mini Kit (Qiagen) and treated with DNase I (Qiagen). The pull-down assays were performed using the Pierce™ Magnetic RNA-Protein Pull-Down Kit (Thermo Fisher, Cat. 20164), in accordance with manufacturer’s instructions. Purified RNAs were biotin-labeled with the Pierce RNA 3′ End Desthiobiotinylation Kit (Thermo Fisher, Cat. 20163). Positive control, negative control, and biotinylated RNAs were mixed and incubated with BGC823 cell lysates. Then, magnetic beads were added to each binding reaction and incubated at room temperature. Finally, the beads were washed and the eluted proteins were detected by Western blot analysis.

### Statistical analysis

All of the statistical analyses were performed using SPSS 17.0 (IBM, IL, USA) software. Student’s *t* test, *χ* ^2^ test, or Wilcoxon’s test were used to analyze the significance of the differences between groups. The overall survival (OS) and disease-free survival (DFS) dates were analyzed by the Kaplan–Meier method with the log-rank test. Pearson correlation analyses were used to investigate the correlation between AGAP2-AS1 and P21 (CDKN1A) or E-cadherin expression. Two-sided *P* values were calculated and those less than 0.05 were considered significant.

## Results

### AGAP2-AS1 is upregulatd in the GC tissues and associated with poor prognosis

**Fig. 1:**
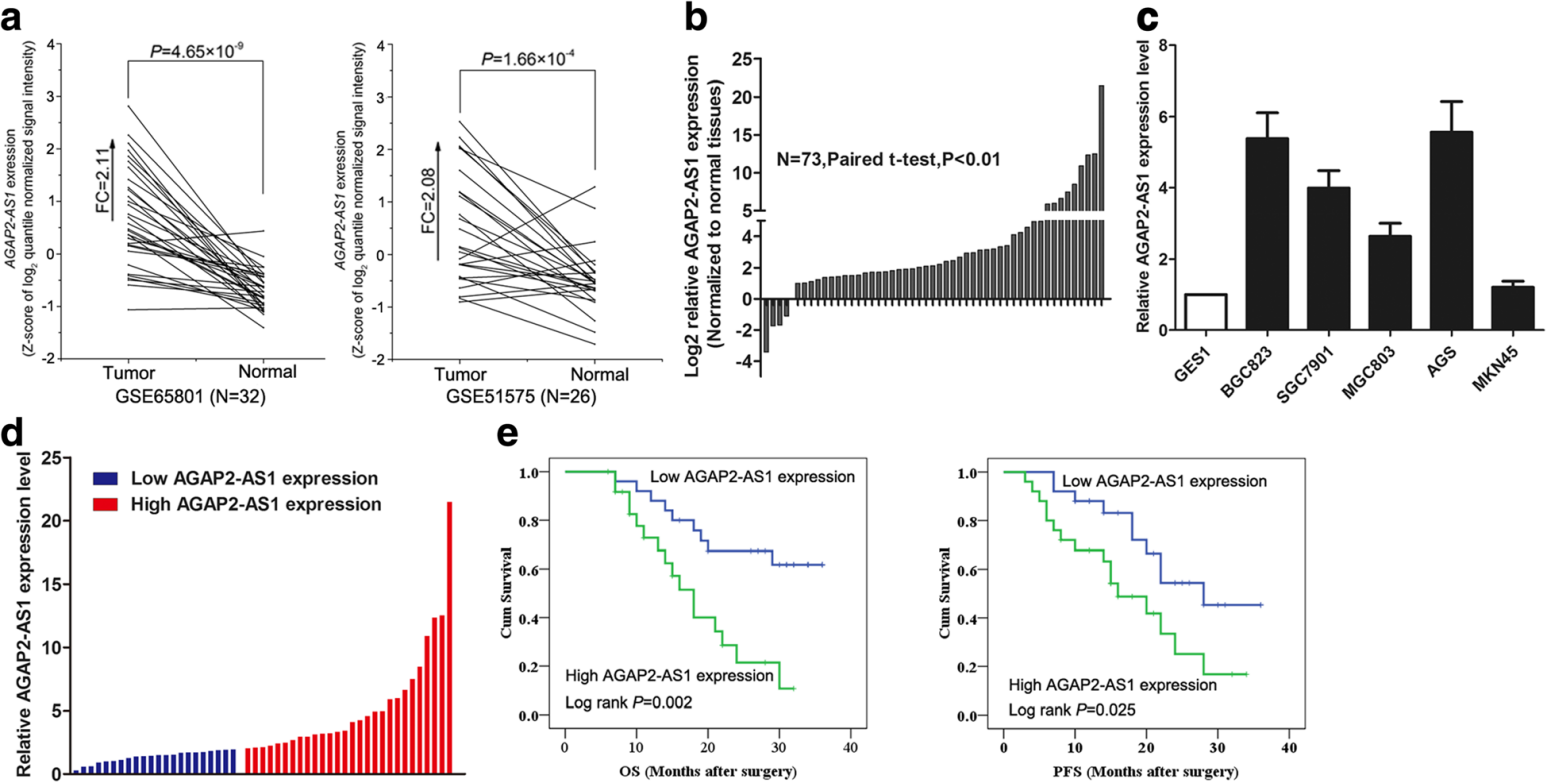
AGAP2-AS1 is overexpressed in the human GC tissues and cells. **a** Data mining of AGAP2-AS1 expression levels in the GC tissue samples from gene profiling (GSE51575 and GSE65801). **b** qRT-PCR analysis of AGAP2-AS1 level in the 50 paired GC tissues and adjacent nontumor tissues. AGAP2-AS1 level was normalized to GAPDH expression. **c** qRT-PCR analysis of AGAP2-AS1 expression in the GC cell lines BGC823, MGC803, SGC7901, AGS, and MKN45 and the normal gastric cell line GSE1. AGAP2-AS1 level was normalized to the GAPDH level. **d** GC patients were divided into two groups according to AGAP2-AS1 expression profiles. The median fold change was used as the threshold. **e** Kaplan–Meier overall and disease-free survival analyses were used to investigate the relationship between AGAP2-AS1 expression and GC patient survival. *P < 0.05, **P < 0.01

To determine the expression pattern of AGAP2-AS1 in the human GC tissues, we first analyzed its expression in two public gene profiling datasets (GSE65801 [21] and GSE51575 [22]) from Gene Expression Omnibus (GEO) database. The analysis results showed that AGAP2-AS1 was highly expressed in the human GC tissues (Fig. 1a). Then, we examined the AGAP2-AS1 expression level in a cohort of the 50 paired GC and nontumor tissues to validate the analysis results. Consistent with these results, we also found that AGAP2-AS1 expression was upregulated in the human GC tissue samples (Fig. 1b). Simultaneously, we determined the expression level of AGAP2-AS1 in GC cell lines (BGC823, SGC7901, MGC803, AGS, and MKN45) and the GES1 cells, an immortalized, normal human gastric cell line, using qRT-PCR. Compared with the level in the GES1 cells, AGAP2-AS1 exhibited higher expression levels in GC cell lines (Fig. 1c). Collectively, these results indicate that AGAP2-AS1 is upregulated in GC.

The GC patients were divided into groups with high (*n* = 25, fold change ≥ median) and low AGAP2-AS1 expression (*n* = 25, fold change ≤ median) to investigate the relationship between this variable and clinicopathology in such patients (Fig. 1d). The statistical analysis showed that a higher AGAP2-AS1 level was associated with larger tumors (*P* = 0.010), advanced pathological stage (*P* = 0.001), and lymph node metastasis (*P* = 0.022). However, AGAP2-AS1 expression level was not related to other factors including gender (*P* = 0.776) and age (*P* = 0.567) in GC (Table 1). Moreover, Kaplan–Meier survival analysis revealed that patients with higher AGAP2-AS1 levels had shorter OS and PFS than those with lower AGAP2-AS1 levels (Fig. 1e).

**Table 1:** Correlation between AGAP2-AS1 expression and clinicopathological characteristics of gastric cancer patients (*n* = 50)

Characteristics		AGAP2-AS1 expression		*P* value
		Low	High	
Age	<50	9	12	0.567
	>50	16	13	
Gender	Male	13	15	0.776
	Fmale	12	10	
location	Distal	11	7	0.338
	Middle	8	13	
	Proximal	6	5	
Tumor size	<5 cm	18	8	0.010^*^
	>5 cm	7	17	
Histologic	Well	3	2	0.011^*^
	Moderately	13	3	
	Poorly	6	16	
	Undifferentiated	3	4	
Lymphatic metastasis	NO	18	9	0.022^*^
	YES	7	16	
TNM stages	I	7	0	0.001^*^
	II	11	3	
	III	7	19	
	IV	0	3	

*Overall *P* < 0.05

### SP1 activated AGAP2-AS1 expression in the GC cells

**Fig. 2:**
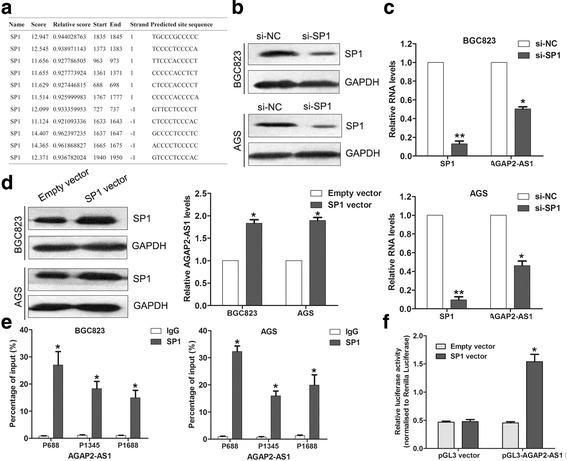
SP1 activates AGAP2-AS1 expression in GC cells. **a** JASPR online prediction of SP1 binding sites in the AGAP2-AS1 promoter regions. **b** Western blot analysis of SP1 protein levels in the AGS and BGC823 cells after transfection with SP1 siRNA. **c** qRT-PCR analysis of SP1 and AGAP2-AS1 expression in AGS and BGC823 cells after transfection with SP1 siRNA. **d** Western blot and qRT-PCR analyses of SP1 and AGAP2-AS1 expression in the AGS and BGC823 cells after transfection with SP1 vector or empty vector. **e** ChIP-qPCR analysis of SP1 occupancy in the AGAP2-AS1 promoter regions in the BGC823 and AGS cells. IgG was used as a negative control. **f** Luciferase reporter analysis of luciferase activity in the HEK293 cells cotransfected with pGL3-AGAP2-AS1 and SP1 vector or an empty vector. *P < 0.05, **P < 0.01

Increasing evidence has revealed that several key transcription factors (TFs) and epigenetic regulators also contribute to lncRNA dysregulation in the human cancer cells, such as p53 [23], E2F1 [24], and EZH2 [25]. Although the above findings and a previous study have shown that AGAP2-AS1 is overexpressed in the human NSCLC and GC tissues, the factors involved in AGAP2-AS1 dysregulation remained unclear. Using the online TF prediction software JASPAR, we found that there are several SP1 binding sites in the AGAP2-AS1 promoter regions (Fig. 2a). Moreover, knockdown of SP1 in the BGC823 and AGS cells by transfection with siRNA decreased AGAP2-AS1 expression (Fig. 2b, c), while ectopic overexpression of SP1 promoted AGAP2-AS1 expression (Fig. 2d). Furthermore, we designed three paired primers covering the promoter regions containing potential SP1 binding sites and performed ChIP assays to evaluate whether SP1 could bind to these sites. The results showed that SP1 could bind to all of these promoter regions of AGAP2-AS1 (Fig. 2e). In addition, the promoter region (2000 bp) of AGAP2-AS1 was inserted into a PGL3 luciferase reporter vector, and Dual-Luciferase Reporter analysis showed that SP1 could bind to this region and activate luciferase (Fig. 2f). These results indicated that AGAP2-AS1 upregulation in GC may be activated partly by SP1.

### Knockdown of AGAP2-AS1 inhibits GC cell proliferation and induced cell cycle arrest

**Fig. 3:**
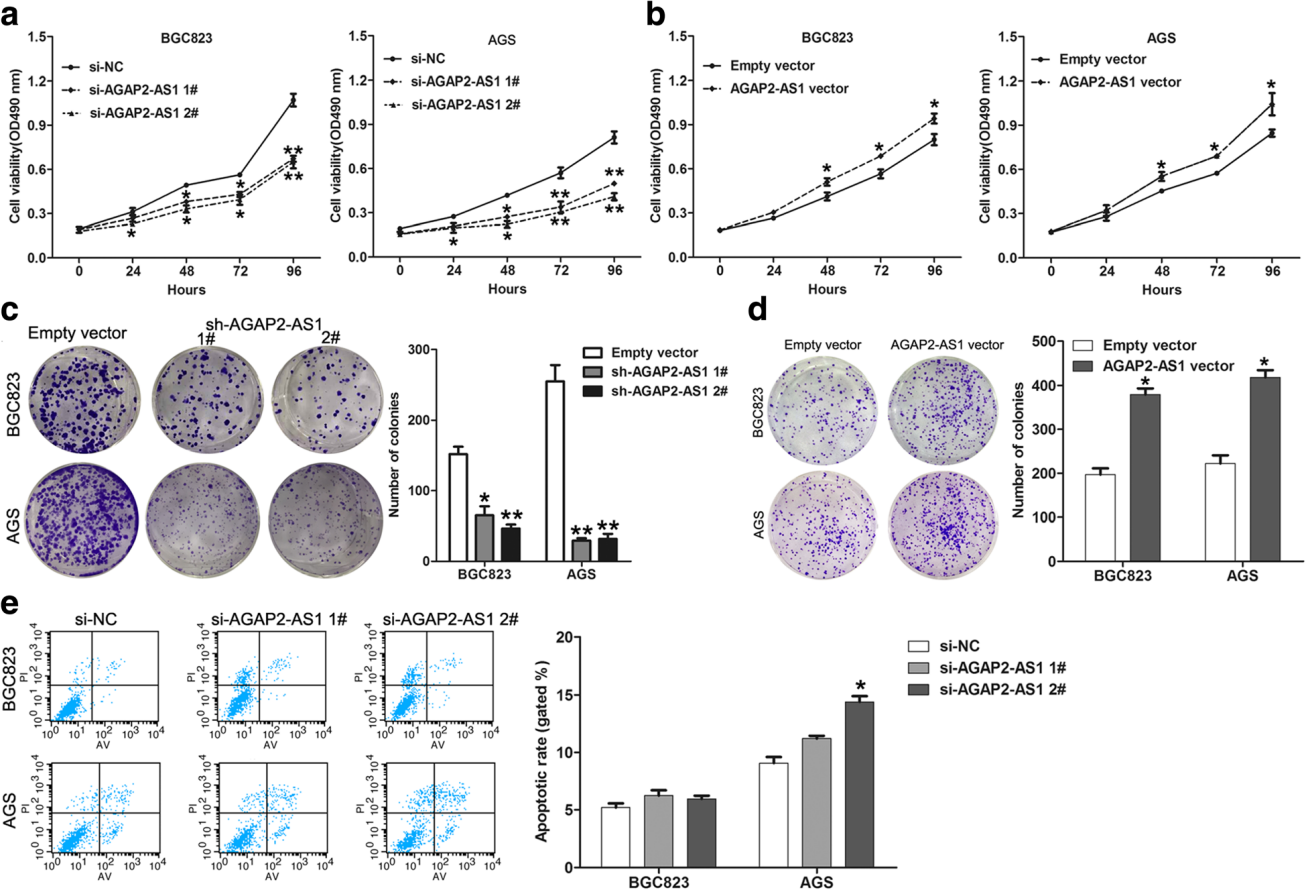
The effect of AGAP2-AS1 on cell proliferation and cell cycle progression. **a** MTT assay analysis of the growth curves for the BGC823 and AGS cells after transfection with AGAP2-AS1 siRNA or negative control. **b** MTT assay analysis of the growth curves for the BGC823 and AGS cells after transfection with AGAP2-AS1 vector or empty vector. **c**, **d** Analysis of the colony formation ability of the BGC823 and AGS cells after transfection with AGAP2-AS1 siRNA, vector, or negative control and empty vector. **e** Flow cytometry cell apoptosis assays were used to analyze the apoptosis of the BGC823 and AGS cells 48 h after transfection with si-AGAP2-AS1. *AV* annexin V. The rate of apoptosis was represented by the proportion of annexin V-positive cells. *P < 0.05, **P < 0.01

As the BGC823 and AGS cells had relative higher AGAP2-AS1 expression levels than that in other cell lines, we thereby chose these two cell lines for further investigation. To evaluate the possible biological function of AGAP2-AS1 in the GC cells, we transfected the BGC823 and AGS cells with two different siRNAs or shRNA vector against AGAP2-AS1, both of which could effectively knock down AGAP2-AS1 expression. We also overexpressed AGAP2-AS1 by transfection with an AGAP2-AS1 overexpression vector (Additional file 2: Figure S1a). Curves of the growth in these experiments, as detected by MTT assays, showed that AGAP2-AS1 knockdown impaired BGC823 and AGS cell growth, while AGAP2-AS1 overexpression promoted the proliferation of these cells (Fig. 3a, b). Consistent with these MTT assay results, knockdown of AGAP2-AS1 drastically inhibited the colony formation ability of the GC cells, while AGAP2-AS1 overexpression enhanced this ability (Fig. 3c, d). To determine the mechanisms underlying the growth suppression after AGAP2-AS1 knockdown, we assessed the effect of this on apoptosis. However, the results of flow cytometry analysis showed that AGAP2-AS1 downregulation had no effect on the apoptosis of the BGC823 cells, while siRNA 2# treatment increased the rate of apoptosis of the AGS cells by about 6% (Fig. 3e).

**Fig. 4:**
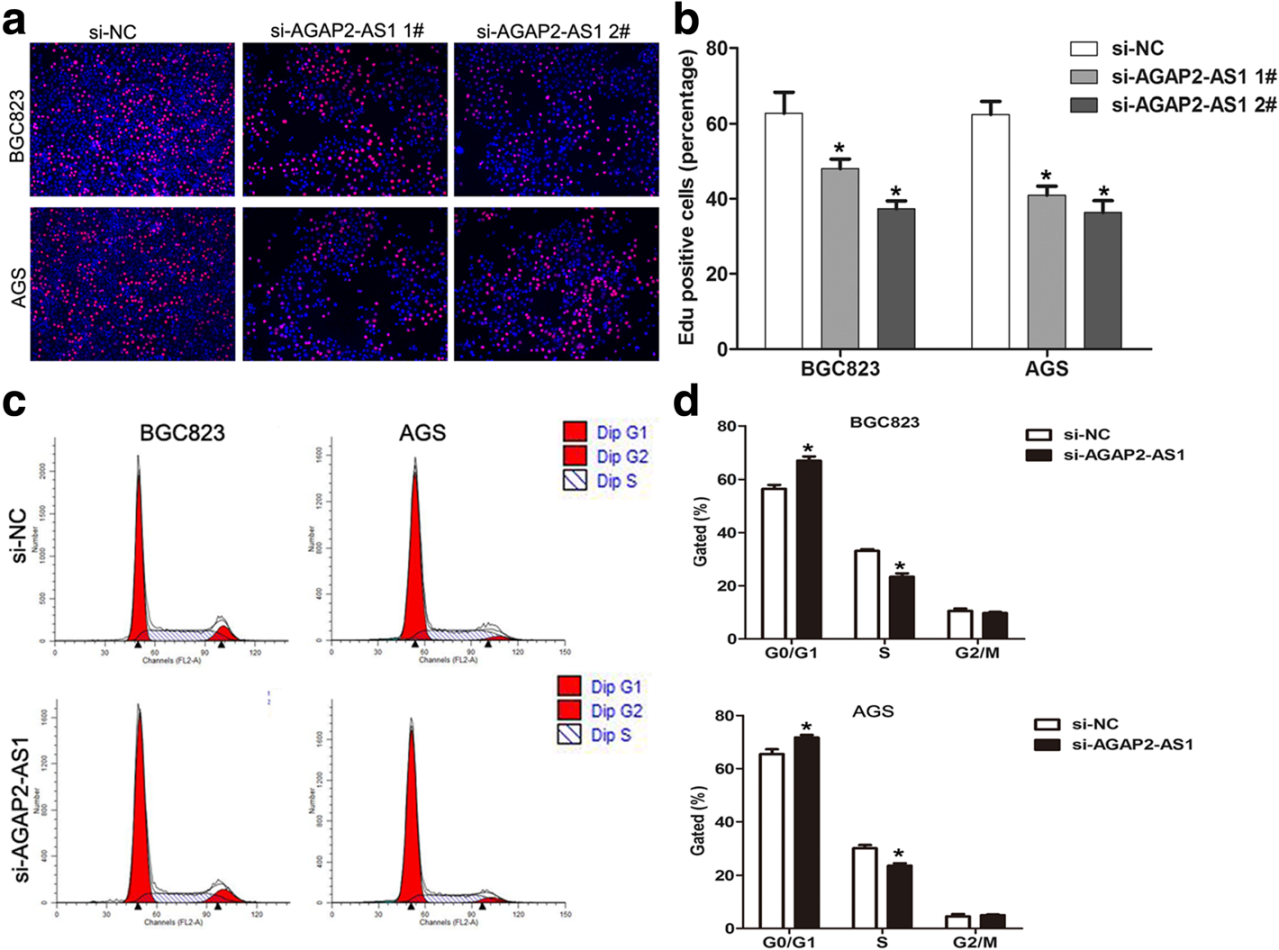
The effect of AGAP2-AS1 on cell cycle progression. **a**, **b** EdU staining assays were performed to determine the growth of the BGC823 and AGS cells 48 h after transfection with si-AGAP2-AS1. **c**, **d** BGC823 and AGS cell cycle progression after transfection with AGAP2-AS1 siRNA or negative control was evaluated by flow cytometry by measuring the proportions of the cells in the G_0_/G_1_, S, and G_2_/M phases using PI staining. All experiments were performed in biological triplicates. *P < 0.05, **P < 0.01

To determine whether a change in cell cycle progression was involved in the AGAP2-AS1-mediated regulation of cell proliferation, we performed EdU staining assays. The results of these assays showed that the rate of the EdU-positive cells was reduced in AGAP2-AS1-downregulated BGC823 and AGS cells (Fig. 4a and b). In addition, the results of flow cytometry analysis showed that AGAP2-AS1 downregulation increased the proportion of G_0_/G_1_ phase cells and decreased the proportion of S phase cells (Fig. 4c, d). These findings indicate that AGAP2-AS1 could promote the cell cycle progression of the GC cells.

### AGAP2-AS1 promotes cell migration and invasion in GC

**Fig. 5:**
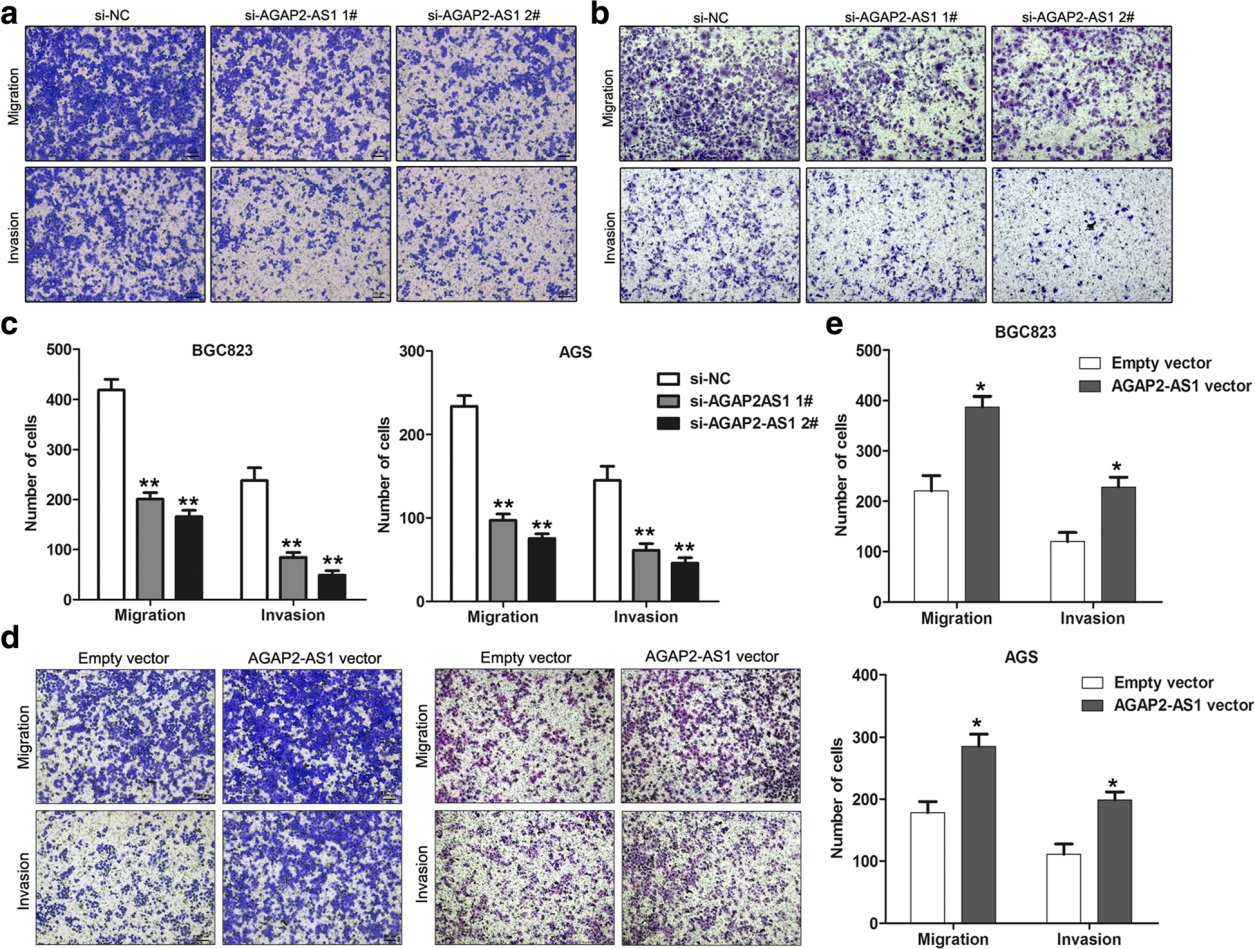
AGAP2-AS1 promotes cell migration and invasion in GC. **a**–**c** The migratory and invasive abilities of the BGC823 and AGS cells after transfection with AGAP2-AS1 siRNA or NC were assessed using Transwell assays. **d**, **e** The migratory and invasive abilities of the BGC823 and AGS cells after transfection with AGAP2-AS1 vector or empty vector were evaluated using Transwell assays. *P < 0.05, **P < 0.01

To further determine whether AGAP2-AS1 is associated with the progression of GC, we analyzed its effect on the migration and invasion of the BGC823 and AGS cells. Using a Transwell assay, we found that the BGC823 and AGS cell migration and invasion were significantly impaired after the knockdown of AGAP2-AS1 (Fig. 5a–c). Conversely, AGAP2-AS1 overexpression in these cells promoted cell migration and invasion (Fig. 5d, e). Taken together, these findings indicate that AGAP2-AS1 has important roles in GC progression.

### AGAP2-AS1 downregulation suppresses GC cell tumorigenesis in vivo

**Fig. 6:**
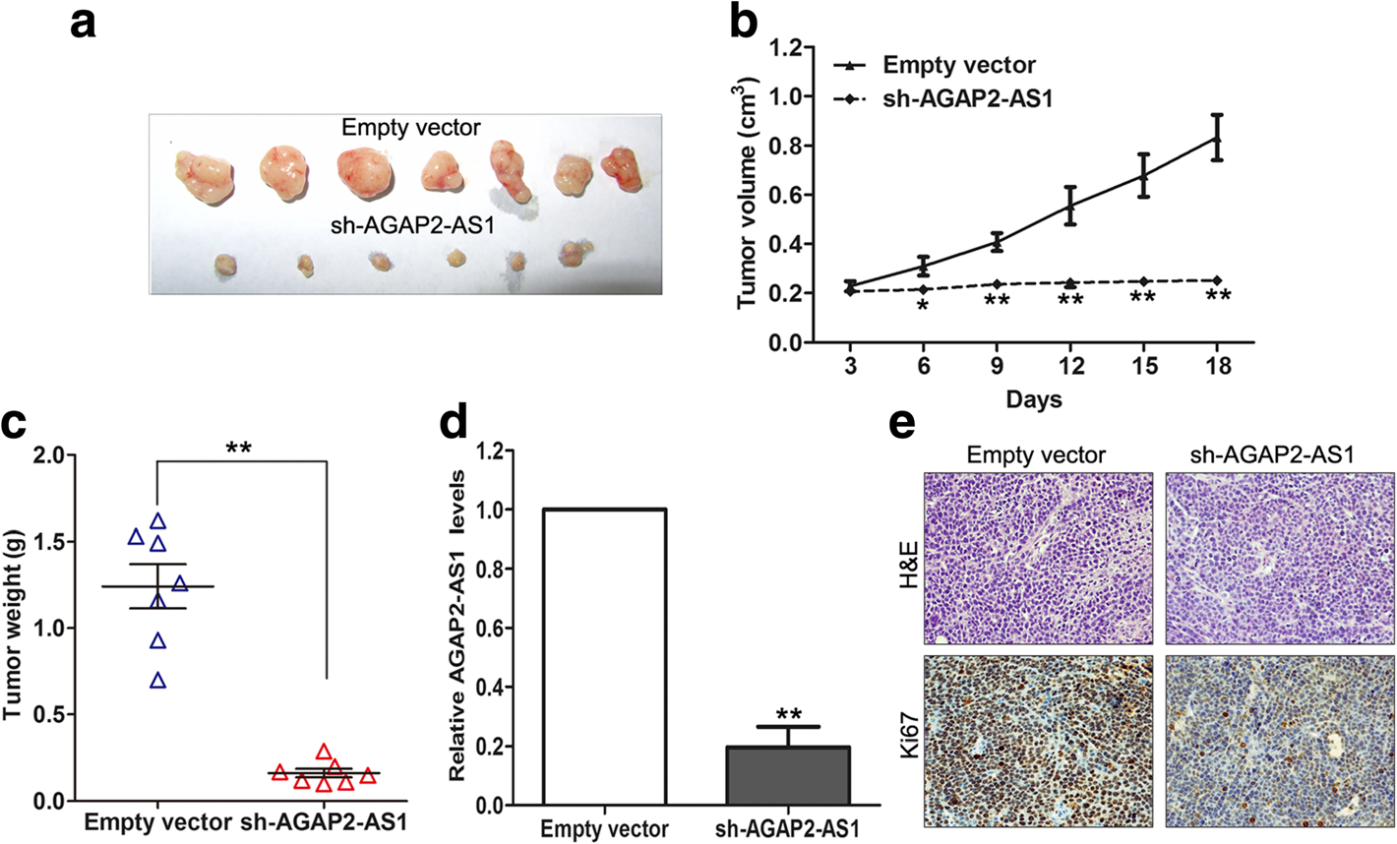
AGAP2-AS1 knockdown inhibits GC cell tumor growth in vivo. **a** The BGC823 cells with stable knockdown of AGAP2-AS1 were used for the in vivo tumorigenesis assays. The tumors formed from the BGC823 cells with AGAP2-AS1 knockdown and the control cells in nude mice are shown. **b** The tumor growth curves were measured 3 days after the injection of the BGC823 cells once the tumor had formed, and the volume was calculated every 3 days. **c** Tumor weights in the sh-AGAP2-AS1 and control groups are presented. **d** qRT-PCR analysis of AGAP2-AS1 expression levels in the tumor tissues formed from the AGAP2-AS1-downregulated cells or control cells. **e** Tumors formed from sh-AGAP2-AS1-transfected BGC823 cells showed lower Ki67-positive signals than tumors formed from the control cells. *Upper*; hematoxylin & eosin staining, *Lower* Ki67 immunostaining. *P < 0.05, **P < 0.01

To verify the above findings obtained in vitro, we constructed the BGC823 cells stably expressing sh-AGAP2-AS1 or negative control by transfection with shRNA vectors. We then injected the control cells and BGC823 cells with stable knockdown of AGAP2-AS1 into nude mice to evaluate whether AGAP2-AS1 affects GC cell tumorigenesis in vivo. The tumors formed by AGAP2-AS1-silenced cells clearly grew much slower than those formed by the control cells (Fig. 6a, b). Moreover, the tumors from the AGAP2-AS1-knockdown group were significantly lighter in weight than those in the control group (Fig. 6c). qRT-PCR analysis of the tumor tissues from the AGAP2-AS1-knockdown and control groups showed that AGAP2-AS1 expression was significantly downregulated in the former group (Fig. 6d). Furthermore, immunohistochemical analysis revealed that the tumors formed by the AGAP2-AS1-knockdown cells exhibited lower Ki-67 staining than those formed by the control cells (Fig. 6e).

### P21 and E-cadherin are targets of AGAP2-AS1 in the GC cells

**Fig. 7:**
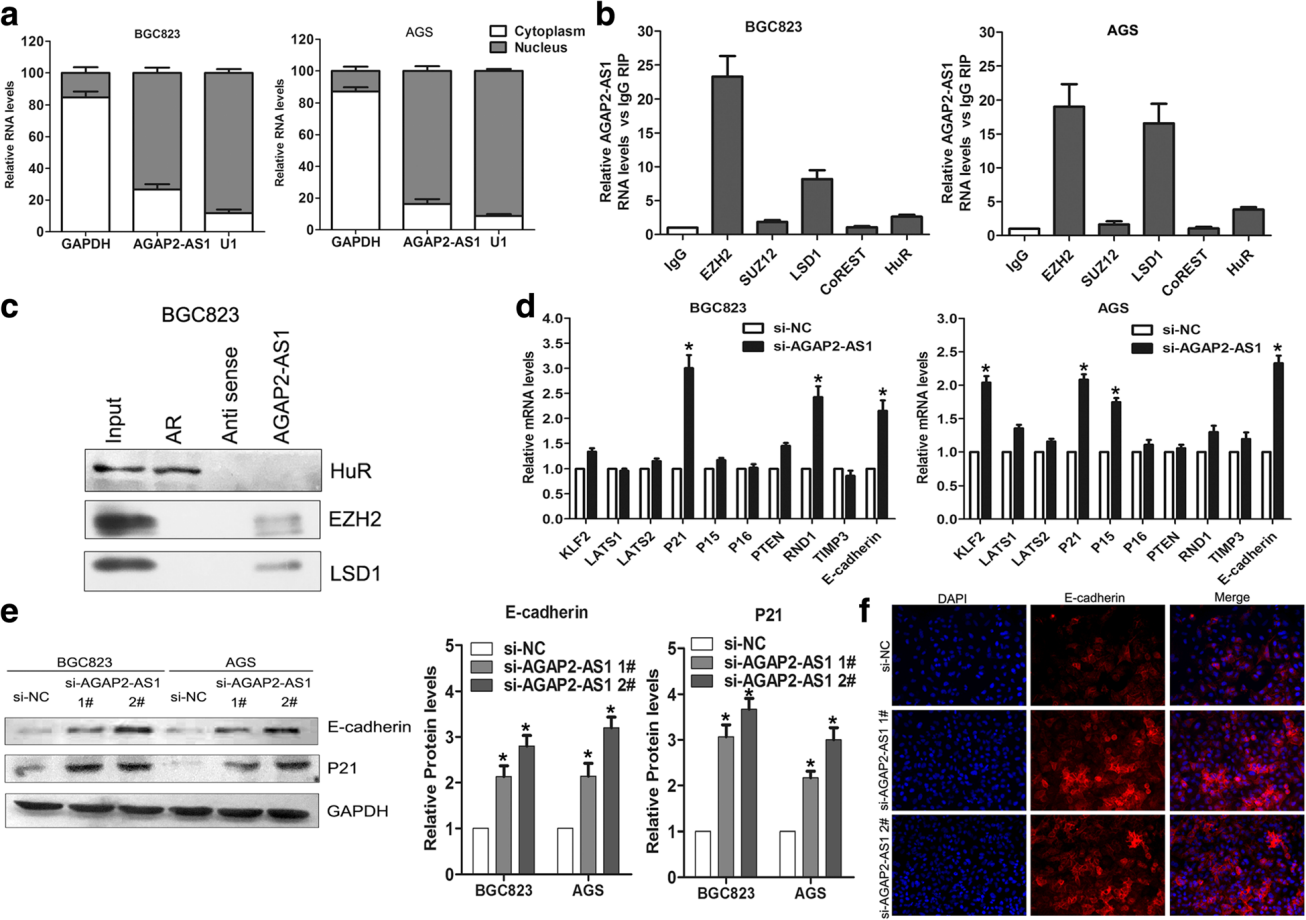
AGAP2-AS1 interacting with EZH2 and LSD1 in the GC cells. **a** The distribution of AGAP2-AS1 levels in the cytoplasmic or nuclear fraction of GC cell lines was determined by qRT-PCR. U1 was used as a nuclear control; GAPDH was used as a cytoplasmic control. **b** The AGAP2-AS1 RNA levels in EZH2, LSD1, SUZ12, CoREST, and HuR immunoprecipitates were determined by qRT-PCR, and data are presented as fold enrichment relative to IgG immunoprecipitates. **c** EZH2 and LSD1 protein levels in immunoprecipitates with AGAP2-AS1 RNA were determined by Western blot. HuR protein immunoprecipitates with AR RNA were used as a positive control. **d** qRT-PCR analysis of the expression levels of KLF2, LATS1, and LATS2, and others in the BGC823 and AGS cells after transfection with AGAP2-AS1 or negative control siRNAs. **e** Western blot analysis of the protein levels of P21 and E-cadherin in the BGC823 and AGS cells after transfection with AGAP2-AS1 or negative control siRNAs. **f** Immunofluorescence staining analysis of E-cadherin in BGC823 cells after transfection with AGAP2-AS1 or negative control siRNAs. *P < 0.05, **P < 0.01

Previous studies have demonstrated that lncRNAs regulate underlying targets by binding to RNA-binding proteins or functioning as endogenous RNAs competing for microRNAs. To determine the molecular mechanism by which AGAP2-AS1 regulates its targets in the GC cells, we first examined its distribution in these cells. The results of fractionation analysis showed that most of the AGAP2-AS1 RNA is located in the nucleus of the GC cells (Fig. 7a), suggesting that it might regulate targets at the transcriptional level. As such, we performed RIP assays to determine whether there is an interaction between AGAP2-AS1 and some well-known RNA-binding proteins including EZH2, SUZ12, LSD1, CoREST, and HuR. The results showed that AGAP2-AS1 could bind to EZH2 and LSD1 in the GC cells (Fig. 7b). Consistent with this, the RNA pull-down analysis also showed that AGAP2-AS1 RNA could directly interact with EZH2 and LSD1 in the BGC823 cells (Fig. 7c). These results suggest that AGAP2-AS1 can epigenetically suppress underlying targets by interacting with EZH2 and LSD1.

We also examined the expression levels of some important tumor suppressors including LATS1, LATS2, KLF2, and PTEN; cyclin-dependent kinase inhibitors including P21, CDKN2B (P15), and CDKN2A (P16); and cell migration and invasion regulators, including TIMP3 and E-cadherin, in the BGC823 and AGS cells after the knockdown of AGAP2-AS1. The results of qRT-PCR showed that the inhibition of AGAP2-AS1 increased P21, RND1, and E-cadherin expression in the BGC823 cells, while KLF2, P21, and E-cadherin expression was upregulated in the AGAP2-AS1-downregulated AGS cells (Fig. 7d). Hence, we chose P21 and E-cadherin as potential targets of AGAP2-AS1 in the GC cells for further validation. Consistent with the results of qRT-PCR, Western blot analysis showed that P21 and E-cadherin protein levels were increased in the si-AGAP2-AS1-transfected BGC823 and AGS cells (Fig. 7e). Meanwhile, immunofluorescence staining confirmed that the expression level of E-cadherin was increased in the AGAP2-AS1-downregulated BGC823 cells (Fig. 7f). These findings suggest that P21 and E-cadherin might be important underlying targets of AGAP2-AS1 in GC.

### AGAP2-AS1 epigenetically suppresses P21 and E-cadherin expression by interacting with EZH2 and LSD1

**Fig. 8:**
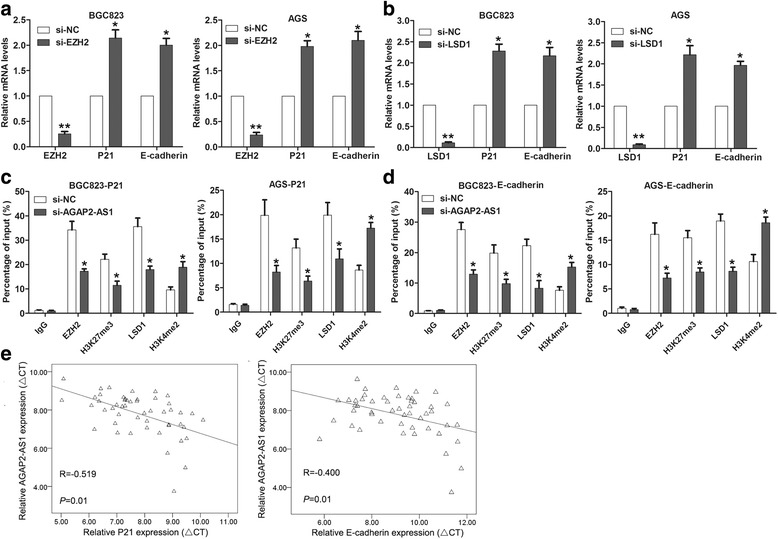
AGAP2-AS1 epigenetically suppresses P21 and E-cadherin by interacting with EZH2 and LSD1. **a**, **b** qRT-PCR analysis of the expression levels of P21 and E-cadherin in the BGC823 and AGS cells after transfection with EZH2, LSD1, or negative control siRNAs. **c**, **d** ChIP-qPCR analysis of EZH2, H3K27me3, LSD1, and H3K4me2 occupancy in the P21 and E-cadherin promoters in the BGC823 and AGS cells after transfection with AGAP2-AS1 or NC siRNA. IgG was used as a negative control. **e** The relationship between AGAP2-AS1 expression and P21/E-cadherin in the GC tissues was analyzed. *P < 0.05, **P < 0.01

To further determine whether AGAP2-AS1 regulates its underlying targets by interacting with EZH2 and LSD1, we determined P21 and E-cadherin expression levels in the BGC823 and AGS cells transfected with EZH2 and LSD1 siRNAs. Interestingly, knockdown of EZH2 and LSD1 also upregulated P21 and E-cadherin expression in the GC cells (Fig. 8a, b, and Additional file 3: Figure S2). To confirm whether EZH2 or LSD1 could bind the promoter regions of P21 and E-cadherin, we performed ChIP analysis. The results showed that EZH2 and LSD1 could bind to the P21 and E-cadherin promoter regions; however, knockdown of AGAP2-AS1 reduced this binding (Fig. 8c, d). Finally, correlation analysis showed that AGAP2-AS1 expression was negatively correlated with P21 and E-cadherin expression in a cohort of the 50 paired GC tissue samples (Fig. 8e).

## Discussion

Recently, RNA sequencing has revealed that hundreds of lncRNAs are dysregulated in several human cancers, indicating that lncRNAs are critical regulators during tumorigenesis and cancer progression [26–28]. Among these lncRNAs, some important ones have been well characterized and their underlying mechanisms in the cancer cells have been revealed. For example, the lncRNA Ras suppressor protein 1 pseudogene 2 (RSU1P2) was shown to be overexpressed in cervical cancer and to play a tumor-promoting role by acting as a ceRNA for microRNA let-7a and regulating the expression of IGF1R, N-myc, and EphA4 [29]. In addition, the expression of the lncRNA GCASPC was significantly lower in the gallbladder cancer (GBC) tissues, and GCASPC overexpression was found to suppress cell proliferation by binding to pyruvate carboxylase [30]. In the present study, we identified that the lncRNA AGAP2-AS1 is upregulated in the human GC tissues and cells by analyzing two independent datasets from GEO; we also validated these findings in a cohort of 50 paired GC and nontumor tissue samples. Moreover, we showed that increased AGAP2-AS1 expression was associated with a poor prognosis and shorter survival time in GC patients. In vitro and in vivo assays revealed that AGAP2-AS1 silencing inhibited cell proliferation, migration, and invasion, while AGAP2-AS1 overexpression promoted cell proliferation and invasion, suggesting that AGAP2-AS1 may have an oncogenic function in gastric tumorigenesis and progression.

Although the expression of numerous lncRNAs has been found to be dysregulated in human cancers, the factors involved in their altered expression pattern in the cancer cells are still not well understood. Interestingly, a few studies have revealed that lncRNA transcription can also be regulated by some TFs and epigenetic regulators. Xu et al. reported that SP1 activated expression of the lncRNA TINCR in the GC cells, resulting in the promotion of cell proliferation [31]. Meanwhile, EZH2 was shown to suppress lncRNA SPRY4-IT1 expression in the NSCLC cells, and DNMT1-mediated DNA methylation was found to lead to MEG3 silencing in gliomas [25, 32]. In this study, using the JASPR online TF binding prediction database, we found that there are several SP1 binding sites in the promoter region of AGAP2-AS1. Then, by applying ChIP and luciferase reporter assays, we determined that SP1 could bind to the AGAP2-AS1 promoter region and activate its transcription. Our findings combined with previous studies suggest that the abnormal activation of transcription factors may play an important role in lncRNA overexpression in the human cancer cells.

Generally, lncRNAs participate in the regulation of cancer cell phenotype by suppressing the expression of tumor suppressors or activating oncogene transcription via diverse mechanisms, such as chromatin remodeling, interacting with histone modification enzymes, and mediating epigenetic alteration, RNA decay, and acting as ceRNAs for specific miRNAs [33–36]. In this study, we performed RIP assays to determine the RNA-binding proteins with which AGAP2-AS1 interacts and found that AGAP2-AS1 could bind to EZH2 and LSD1 in the GC cells. Interestingly, the well-known lncRNA HOTAIR also could interact with EHZ2 and LSD1, which has similar function and is similarly located in the same chromosome as AGAP2-AS1 [37]. Further, qRT-PCR analysis revealed that P21 and E-cadherin might be novel targets of AGAP2-AS1 in the GC cells, and ChIP assays revealed that EZH2 and LSD1 could bind to their promoter regions. Importantly, the knockdown of AGAP2-AS1 reduced the levels of interaction of EZH2 and LSD1 with the P21 and E-cadherin promoters. P21 is a key member of the cyclin-dependent kinase inhibitor family, which controls cell cycle progression. Loss of P21 expression could lead to uncontrolled cell proliferation and the development of cancer, and epigenetic modifications have been found to be involved in decreased P21 expression in the cancer cells [38, 39]. The loss of E-cadherin expression is a hallmark of epithelial–mesenchymal transition, which is implicated in the promotion of cancer cell migration and metastasis [40, 41]. Recent studies have revealed that the epithelial–mesenchymal transition is a potential mechanism by which the cancer cells detach from primary tumors [41]. Therefore, AGAP2-AS1-mediated suppression of P21 and E-cadherin could account for its promotion of cell proliferation, migration, and invasion in GC.

## Conclusions

Taking the obtained findings together, our study shows for the first time that expression of the lncRNA AGAP2-AS1 is upregulated in the GC tissues and cells, and increased AGAP2-AS1 is associated with poor prognosis of GC patients. Knockdown of AGAP2-AS1 exerted tumor-suppressive effects by inhibiting cell proliferation, migration, and invasion. Furthermore, the transcription factor SP1 activated AGAP2-AS1 transcription, and AGAP2-AS1-mediated oncogenic effects occurred partially through epigenetic suppression of P21 and E-cadherin expression by binding to EZH2 and LSD1. Our findings increase our understanding of the pathogenesis and progression of GC and may facilitate the development of lncRNA-directed diagnostics and therapeutics in GC. However, whether AGAP2-AS1 can regulate other targets through different mechanisms was not investigated, so this should be focused on in further studies.

## Additional files

Additional file 1: Table S1. Primers, siRNAs and shRNAs sequence. (XLSX 13 kb)
Additional file 2: Figure S1. Modulation of AGAP2-AS1 expression in the BGC832 and AGS cells. (a) qRT-PCR analysis of AGAP2-AS1 expression levels in the BGC823 and AGS cells after transfection with AGAP2-AS1 or negative control siRNAs. (b) qRT-PCR analysis of AGAP2-AS1 expression levels in the BGC823 and AGS cells after transfection with sh-AGAP2-AS1 vector or empty vector. (c) qRT-PCR analysis of AGAP2-AS1 expression levels in the BGC823 and AGS cells after transfection with AGAP2-AS1 vector or empty vector. **P* < 0.05, ***P* < 0.01. (TIF 1489 kb)
Additional file 3: Figure S2. Knockdown of EZH2 and LSD1 expression in the BGC823 and AGS cells. (a, b) Western blot analysis of the expression levels of EZH2 in the BGC823 and AGS cells after transfection with EZH2 or negative control siRNAs. (c, d) Western blot analysis of the expression levels of LSD1 in BGC823 and AGS cells after transfection with LSD1 or negative control siRNAs. **P* < 0.05, ***P* < 0.01. (TIF 1553 kb)

## Abbreviations

ceRNA: Competing endogenous RNA
DFS: Disease-free survival
GC: Gastric cancer
lncRNA: Long noncoding RNA
OS: Overall survival
qRT-PCR: Quantitative real-time polymerase chain reaction
TF: Transcription factor
