# Supplementary figures and images for: Long noncoding AGAP2-AS1 is activated by SP1 and promotes cell proliferation and invasion in gastric cancer
Source: J Hematol Oncol. 2017 Feb 16;10:48. doi: 10.1186/s13045-017-0420-4 (PMC5314629; doi:10.1186/s13045-017-0420-4)

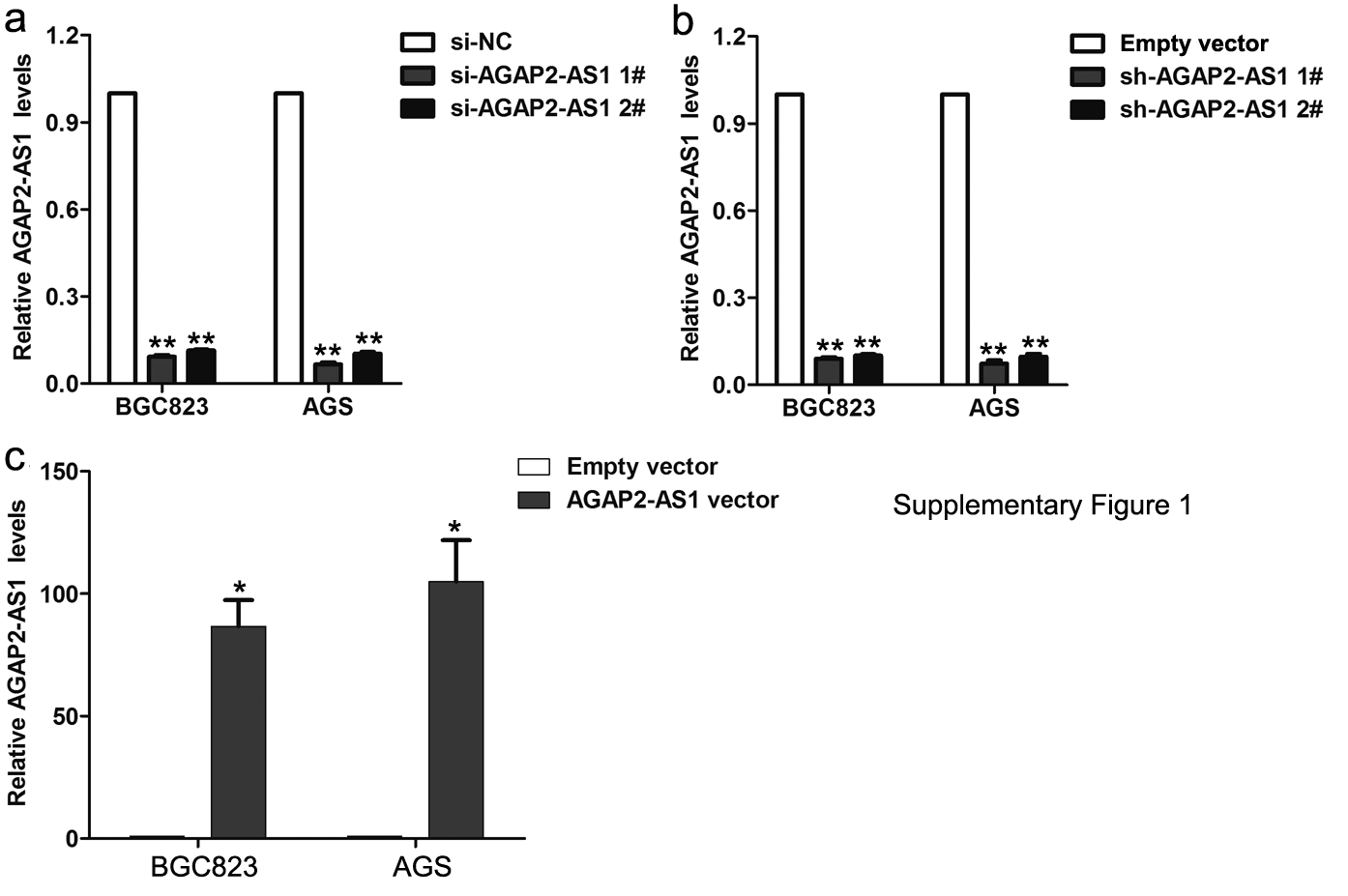

Supplement: Additional file 2: Figure S1. — Modulation of AGAP2-AS1 expression in the BGC832 and AGS cells. (a) qRT-PCR analysis of AGAP2-AS1 expression levels in the BGC823 and AGS cells after transfection with AGAP2-AS1 or negative control siRNAs. (b) qRT-PCR analysis of AGAP2-AS1 expression levels in the BGC823 and AGS cells after transfection with sh-AGAP2-AS1 vector or empty vector. (c) qRT-PCR analysis of AGAP2-AS1 expression levels in the BGC823 and AGS cells after transfection with AGAP2-AS1 vector or empty vector. *P < 0.05, **P < 0.01. (TIF 1489 kb) [file 13045_2017_420_MOESM2_ESM.tif]

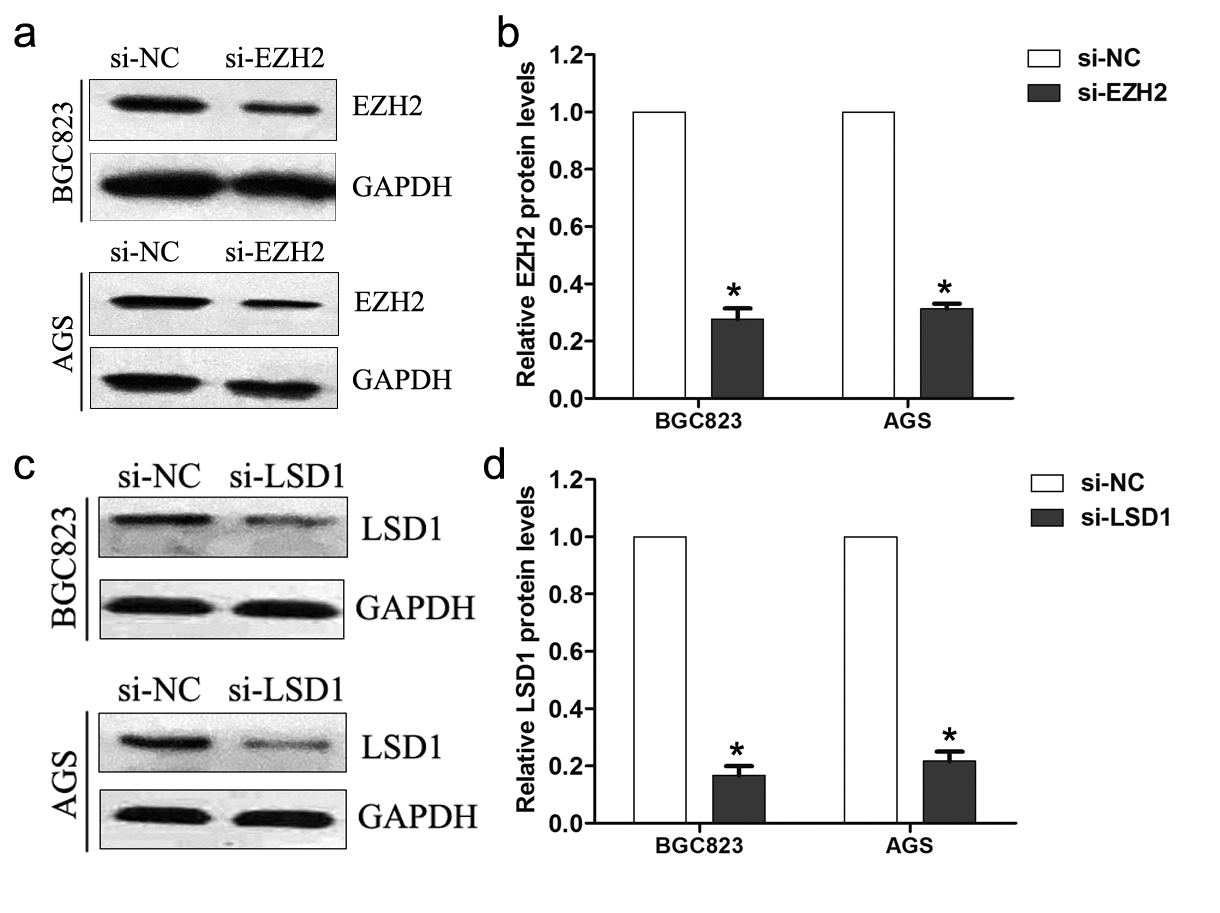

Supplement: Additional file 3: Figure S2. — Knockdown of EZH2 and LSD1 expression in the BGC823 and AGS cells. (a, b) Western blot analysis of the expression levels of EZH2 in the BGC823 and AGS cells after transfection with EZH2 or negative control siRNAs. (c, d) Western blot analysis of the expression levels of LSD1 in BGC823 and AGS cells after transfection with LSD1 or negative control siRNAs. *P < 0.05, **P < 0.01. (TIF 1553 kb) [file 13045_2017_420_MOESM3_ESM.tif]
